# Gamifying hospital site tours for medical students

**DOI:** 10.1111/medu.15665

**Published:** 2025-03-05

**Authors:** Harry Kingsley‐Smith, Alice Ditchfield, Matthew Rollin

## 1 WHAT PROBLEMS WERE ADDRESSED?

Commencing hospital‐based placements can be stressful for medical students. Inductions aim to streamline their transition into this new learning environment. Gruppen and colleagues have conceptualised healthcare learning environments within two dimensions: the material and the psychosocial.^1^ Problems arise in each dimension. Materially, the physical spaces of hospitals can be difficult to navigate. Socially, large cohorts reduce the likelihood of students being familiar with their placement peers. Furthermore, psychologically, educators aim for students to progressively develop autonomy.

## 2 WHAT WAS TRIED?

We gamified a 1‐hour site tour to promote peer‐to‐peer collaboration and independent problem solving. The tour was for medical students undertaking their first‐ever hospital placement, at St. Mary's Hospital, a large tertiary care hospital in Paddington, London, UK. Thirty‐eight first‐year students participated.

We divided students into their placement groups and tasked them with finding relevant hospital locations. Locations included a group's assigned ward, the emergency department, clinics and occupational health. We placed lettered signs outside these locations to create an anagram. We asked groups to take a photograph with each sign to verify visits, while avoiding photographing others, sensitive information or entering busy/critical areas. Given the local statues of Paddington Bear, we related the anagram solution to him. We awarded a prize to the first group to visit each location and solve the anagram.

We asked students to complete an anonymous post‐tour Qualtrics™ questionnaire, featuring a 5‐point pictorial scale reflecting their tour experience (from ‘very unhappy’ to ‘very happy’). The questionnaire also asked if they preferred the gamified tour to a staff‐led tour and to explain their preference.

## 3 WHAT LESSONS WERE LEARNED?

Our findings, from 24 participants (62%), show that students were generally happy with the gamified tour, with the median slider score being 4/5. Furthermore, students preferred the idea of the gamified self‐led tour (*n* = 17). They broadly described the tour as fun (n = 9), engaging (*n* = 4) and a good ‘icebreaker’ for peers to become acquainted (*n* = 5).

Conversely, six students would have preferred a staff‐led tour, finding it tiring (*n* = 2) and difficult to find areas (*n* = 4). One student felt the activity was ‘immature and inappropriate’ and another suggested not referencing Paddington Bear. We believe changing the anagram solution would address these comments.

Most notably, another student felt it was ‘disrespectful’ to be carrying out the activity in areas ‘where people are in obvious distress.’ Unfortunately, some signs had reportedly fallen down/disappeared from certain locations, which might have led students to enter areas looking for them. To our knowledge, no staff or patients complained about the tour. Interestingly, two students conjectured that other groups had removed these signs, perhaps showing an inappropriate engagement with the task. Hence, we suggest a heavy emphasis on staying outside of critical areas and the use of sturdier signage for such tours.

Overall, the gamified tour was engaging, positively received and successfully orientated students to the hospital. In a small way, it acted as a peer‐to‐peer icebreaker activity and encouraged collaborative problem solving when transitioning to a new learning environment.

## CONFLICT OF INTEREST STATEMENT

None to declare.

## Data Availability

The data that support the findings of this study are available from the corresponding author upon reasonable request.
